# Mitral Valve Replacement via Right Thoracotomy in a Patient With Marfan Syndrome and Severe Scoliosis After a Prior Bentall Procedure

**DOI:** 10.7759/cureus.90616

**Published:** 2025-08-20

**Authors:** Yuya Tsuruta, Akihiro Higashino, Tsuyoshi Taketani, Sumio Miura, Takayuki Ohno

**Affiliations:** 1 Cardiovascular Surgery, Mitsui Memorial Hospital, Tokyo, JPN

**Keywords:** marfan syndrome, mitral valve regurgitation, mitral valve surgery, right thoracotomy, severe scoliosis

## Abstract

Mitral valve surgery is commonly performed through median sternotomy or right mini-thoracotomy, but both approaches can be challenging in patients with thoracic deformities. We report the case of a 37-year-old male patient with Marfan syndrome, severe scoliosis, and a history of prior Bentall procedure for acute type A aortic dissection, who developed heart failure due to severe mitral regurgitation. Given his anatomical complexity and surgical history, neither median sternotomy nor right mini-thoracotomy was deemed feasible. A right anterolateral thoracotomy was selected, and mitral valve replacement with a mechanical valve was completed uneventfully. This case suggests that right thoracotomy remains an alternative for mitral valve surgery in patients with complex anatomy, including severe scoliosis and previous aortic surgery.

## Introduction

The right thoracotomy approach has been performed for several decades as part of the early mitral valve surgery series. However, with the establishment of median sternotomy as the standard approach, this approach was abandoned. Subsequently, although it regained attention in reoperative settings due to the risks associated with resternotomy [1,2], its role has again diminished with the increasing adoption of minimally invasive mitral valve surgery (MIMVS) and the accumulating evidence supporting its efficacy in redo cases [3,4].

Currently, mitral valve surgery is commonly performed via either median sternotomy or right mini-thoracotomy. Median sternotomy provides wide applicability and excellent exposure and, therefore, remains the standard approach in cardiac surgery. In contrast, a right mini-thoracotomy is less invasive and is associated with faster recovery and a lower risk of infection; however, its indications are limited.

While both approaches offer specific advantages, patients with connective tissue disorders associated with thoracic deformities may pose significant challenges, particularly in terms of exposing and visualizing the mitral valve. In certain reoperative situations, such as prior aortic surgery or the presence of patent coronary bypass grafts, the right mini-thoracotomy approach can be technically demanding and necessitate advanced surgical expertise, as severe adhesions increase the risk of graft injury.

In this report, we present the use of right thoracotomy in a patient with Marfan syndrome (MFS), severe scoliosis, and a history of the Bentall procedure for acute type A aortic dissection, highlighting the advantages of this approach in a complex reoperative setting.

## Case presentation

A 37-year-old male with MFS presented with dyspnea and was admitted for heart failure. His medical history included three spinal surgeries for scoliosis and a Bentall procedure for acute type A aortic dissection. Although severe mitral regurgitation (MR) had been previously diagnosed, he remained asymptomatic with preserved left ventricular function and was therefore managed conservatively.

**Figure 1:**
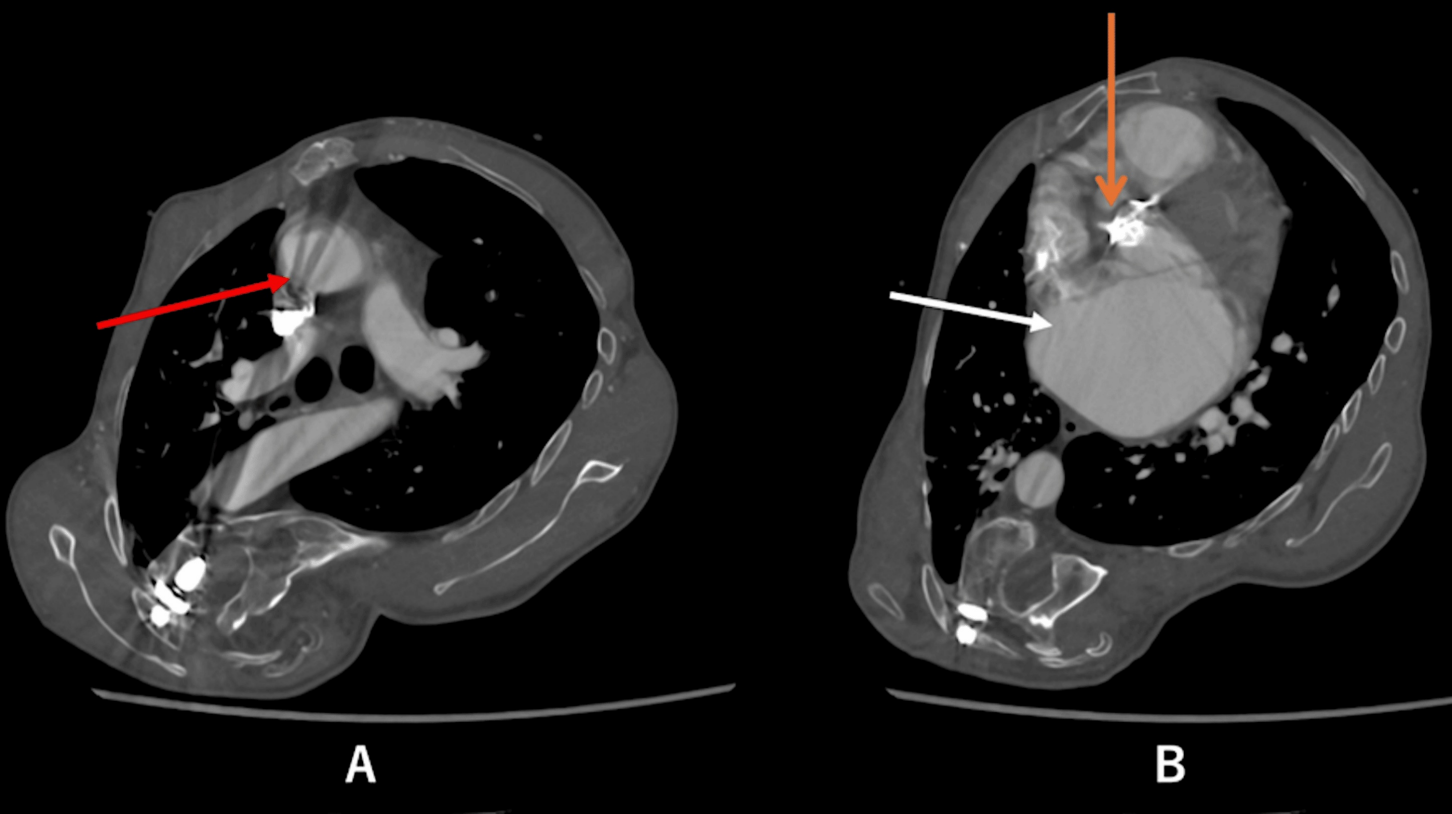
Axial CT images at the level of the second ICS (A) and fourth ICS (B) The orange arrow indicates the trajectory of a median sternotomy, which does not provide adequate exposure for the mitral valve. The white arrow indicates the right thoracotomy approach through the fourth ICS, while the red arrow indicates the second ICS, used for dissection around the aortic graft. ICS: intercostal space

**Figure 2:**
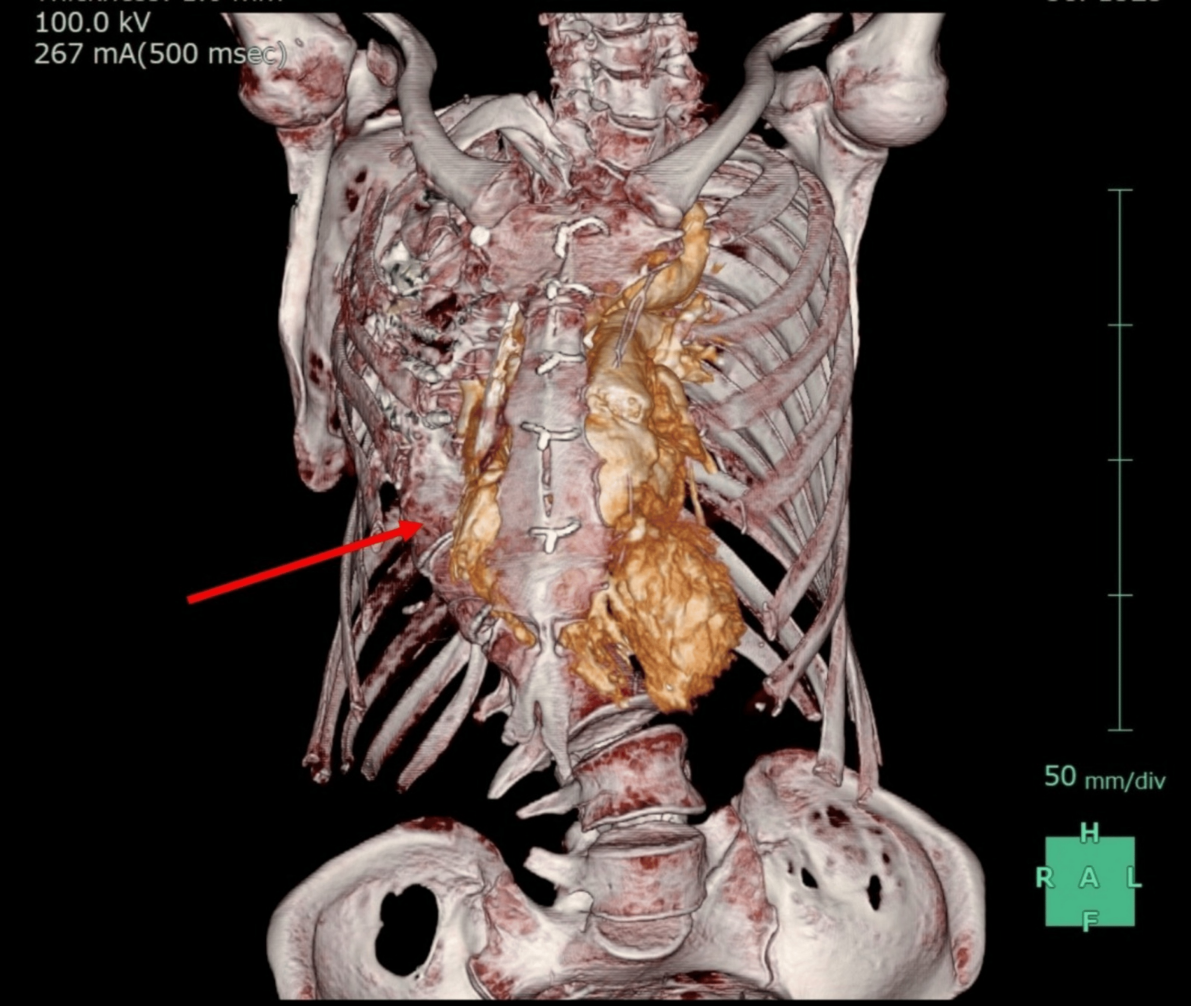
Three-dimensional reconstruction generated from CT imaging The red arrow indicates the presence of severe scoliosis.

Transthoracic echocardiography showed severe MR due to anterior leaflet prolapse. The ejection fraction was 69%, with a left ventricular end-systolic dimension of 22 mm and an end-diastolic dimension of 40 mm. The effective regurgitant orifice area (EROA) was 0.57 cm², and the regurgitant volume measured 96 mL. Preoperative computed tomography (CT) demonstrated extremely severe scoliosis with a Cobb angle of 90°, which resulted in a cardiac displacement within the thoracic cavity (Figures 1, 2).

Mitral valve surgery was indicated due to symptomatic severe MR. However, median sternotomy was deemed technically unfeasible because of the cardiac malposition. A right mini-thoracotomy was also considered inappropriate due to anticipated adhesions around the aortic graft. Consequently, we selected the right thoracotomy approach.

An anterolateral thoracotomy was made through the fourth intercostal space. Dense adhesions were noted below the fifth rib, and an additional incision was made at the second intercostal space to dissect around the prosthetic graft. Cardiopulmonary bypass (CPB) was initiated via the right femoral vessel cannulation. A left heart vent was placed via the right superior pulmonary vein, and the aortic graft was cross-clamped through the second intercostal incision. Antegrade crystalloid cardioplegia was initially administered into the aortic graft, followed by retrograde delivery. The exposure of the mitral valve through the left atriotomy was satisfactory. It showed diffuse prolapse of the anterior leaflet and advanced myxomatous degeneration without chordal rupture. The annulus was mildly dilated and non-calcified. The valve was considered unsuitable for durable repair, so we proceeded with valve replacement. We resected the anterior leaflet and implanted a 31-mm St. Jude Medical mechanical valve (Abbott, IL, USA) in the intra-annular position using pledgeted 2-0 ETHIBOND sutures. Intraoperative transesophageal echocardiography confirmed the absence of residual MR. CPB weaning was smooth. The times of operation, CPB, and cross-clamp were 254, 112, and 63 minutes, respectively. The estimated blood loss was 147 ml.

**Figure 3:**
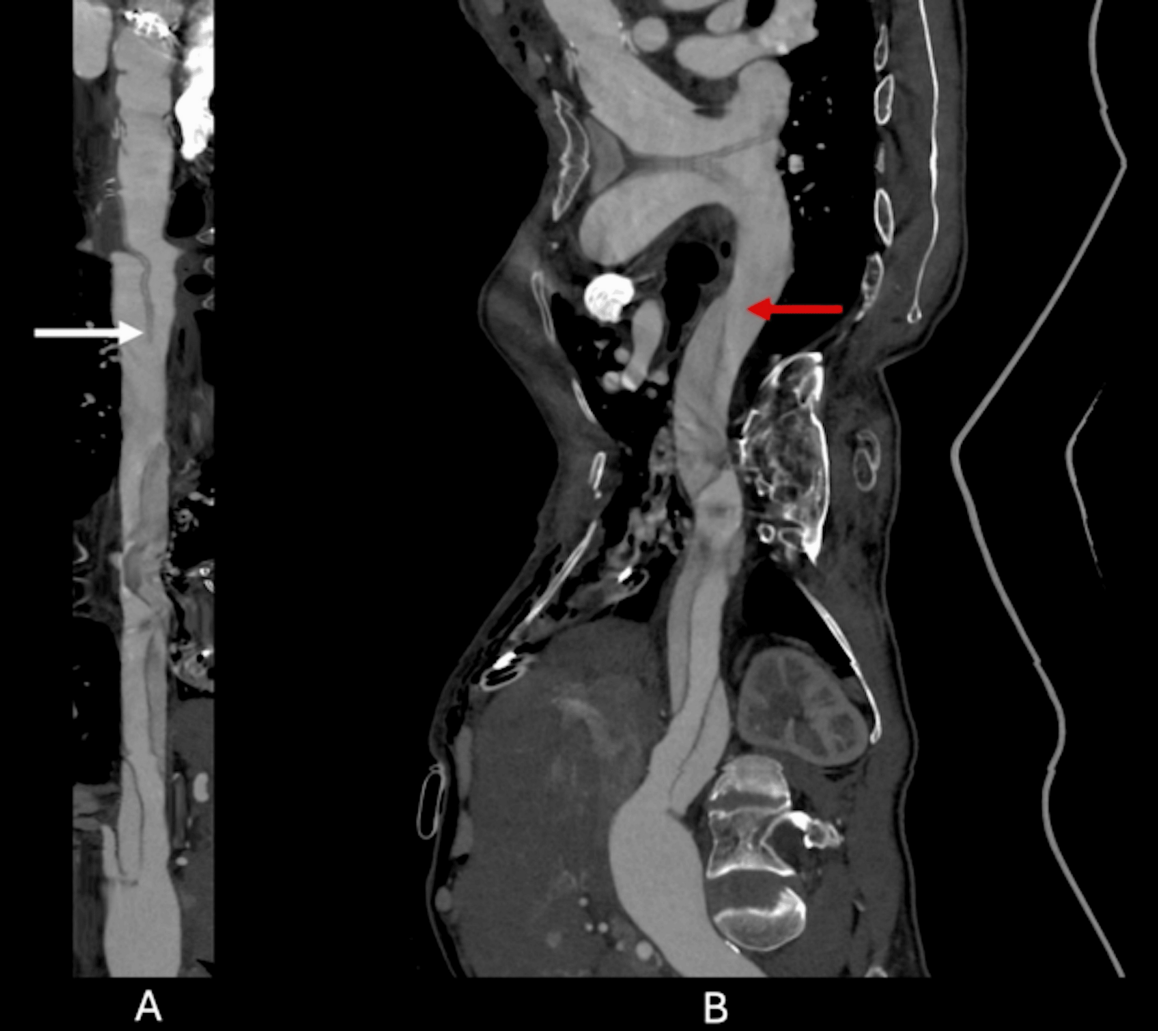
CPR images: stretched view (A) and straightened view (B) The white arrow indicates the old intimal flap from the type A aortic dissection extending to the distal arch. The red arrow indicates the new flap and entry point of the type B aortic dissection, which were unrelated to the mitral valve surgery. CPR: curved planar reconstruction

He was extubated on postoperative day one and remained in the intensive care unit for two days. On postoperative day three, he reported back pain. CT revealed a new acute type B aortic dissection with a primary tear in the proximal descending aorta extending into the abdominal aorta (Figure 3). A maximum descending aortic diameter was 22 mm, and no malperfusion was observed.

Postoperative echocardiography showed trivial transvalvular MR due to the hinge, with no paravalvular leak. The maximum mitral valve velocity was 1.2 m/s, and the mean pressure gradient was 2 mmHg.

Apart from this complication, his postoperative course was uneventful, and he was discharged home on postoperative day 17. At the one-year follow-up, no paravalvular leak or valvular dysfunction was observed, although the descending aortic diameter had increased to 33 mm.

## Discussion

In current clinical practice, mitral valve surgery is primarily performed via median sternotomy or right mini-thoracotomy. However, in patients with complex anatomical challenges, such as those with thoracic deformities or a history of prior cardiac surgeries, these approaches may not be feasible.

Patients with MFS often exhibit skeletal abnormalities, including scoliosis and pectus excavatum. In particular, severe scoliosis can distort thoracic anatomy and mediastinal orientation, rendering median sternotomy technically challenging. In such cases, alternative surgical approaches may provide safer access. Bartolozzi et al. and Bernhardt et al. [5,6] reported successful MIMVS in patients with MFS and severe scoliosis. However, when right thoracotomy is also technically unfeasible, left thoracotomy may represent a viable alternative. Bastidas et al. and LeMaire et al. [7,8] described mitral valve replacement via left thoracotomy in patients with pectus excavatum, in whom leftward displacement of the heart precluded both median sternotomy and right thoracotomy.

Adhesions resulting from prior aortic surgery present additional technical challenges. Dissection around a prosthetic aortic graft during MIMVS is particularly demanding. Although Vallabhajosyula et al. and Arcidi Jr. et al. [3,4] highlighted the advantages of MIMVS in reoperative settings, their cohorts excluded patients who had undergone prior aortic surgery. To avoid dissection near the ascending aorta, Zhang et al. and Romano et al. [9,10] described mitral valve surgery using beating heart or hypothermic ventricular fibrillation techniques in reoperative cases. However, these strategies have not been widely adopted and carry a potential risk of air embolism.

In the present case, we selected a conventional right thoracotomy rather than a minimally invasive approach due to anticipated pleural adhesions and the history of a Bentall procedure. Although the beating heart technique was considered, we believe that secure aortic cross-clamping and controlled cardioplegia represent the safest strategy. While more invasive than MIMVS, this approach avoids the risks associated with resternotomy, such as graft injury and mediastinitis, and offers excellent exposure for mitral valve replacement in patients with complex anatomical and surgical histories.

## Conclusions

In patients with MFS, severe scoliosis is frequently observed, and many have undergone both previous aortic surgery and thoracotomy for scoliosis surgery. In such situations, median sternotomy and right mini-thoracotomy can be technically challenging. Right thoracotomy should be considered a viable and safe alternative for mitral valve surgery in high-risk patients with complex anatomical conditions.
